# *In situ* characterization of stresses, deformation and fracture of thin films using transmission X-ray nanodiffraction microscopy

**DOI:** 10.1107/S1600577523010093

**Published:** 2024-01-01

**Authors:** Gudrun Lotze, Anand H. S. Iyer, Olof Bäcke, Sebastian Kalbfleisch, Magnus Hörnqvist Colliander

**Affiliations:** a MAX IV Laboratory, Lund, Sweden; b LINXS Institute of Advanced Neutron and X-ray Science, Lund, Sweden; c Department of Physics, Chalmers University of Technology, Gothenburg, Sweden; University of Malaga, Spain

**Keywords:** nanodiffraction, stress mapping, *in situ* deformation, nanoindentation, sample environment

## Abstract

The use of hard X-ray transmission nano- and microdiffraction to perform *in situ* stress and strain measurements during deformation has recently been demonstrated and used to investigate many thin film systems. Here a newly commissioned sample environment based on a commercially available nanoindenter is presented, which is available at the NanoMAX beamline at the MAX IV synchrotron. Using X-ray nanoprobes of around 60–70 nm at 14–16 keV and a scanning step size of 100 nm, we map the strains, stresses, plastic deformation and fracture during nanoindentation of industrial coatings with thicknesses in the range of several micrometres, relatively strong texture and large grains. The successful measurements of such challenging samples illustrate broad applicability. The sample environment is openly accessible for NanoMAX beamline users through the MAX IV sample environment pool, and its capability can be further extended for specific purposes through additional available modules.

## Abstract 2

Scanning nanodiffraction mapping was used to map stresses, plastic deformation and cracking of hard coatings during nanoindentation.

## 1. Introduction

In recent years, high-flux hard X-ray probes with beam sizes in the micrometre and sub-micrometre range have become increasingly available at synchrotron sources. Thereby, nanodiffraction is finding more and more applications within the field of materials science (Ice *et al.*, 2011 ▸; Martinez-Criado, 2015 ▸; Schülli & Leake, 2018 ▸). While X-ray nanoprobes enable a multitude of different techniques and applications, we will focus here on scanning diffraction experiments, where extended strain and stress fields, as well as microstructural features and cracking, in hard polycrystalline coatings are mapped. Such coatings are regularly applied for wear resistance and environmental protection in extreme applications, *e.g.* as cutting tools for metal machining. Typically, these coatings are composed of different combinations of transition metal oxides, carbides, nitrides or carbonitrides, with layer thicknesses of the order of several micrometres, deposited on, for example, sintered cemented carbide tool substrates.

Measuring the development of stress and strain distribution in these high-performance coatings during deformation is difficult, due to the limited thickness. Other techniques with suitable spatial resolution either lack stress-measurement capabilities or are limited by surface-only imaging (*e.g.* scanning electron microscopy and related techniques), leading to non-representative measurements, or the need for excessively small samples (*e.g.* transmission electron microscopy based techniques), which leads to relaxation of the important residual stress fields in the coatings. Using scanning nanoprobe X-ray diffraction, it was demonstrated that cross-sectional transmission diffraction of suitably prepared coating samples provided a means to map phase content, texture, and residual strains and stresses in coatings without significant stress relaxation (Keckes *et al.*, 2012 ▸; Stefenelli *et al.*, 2013 ▸). By producing slices of the coating/substrate, with the coating still attached and a large enough width to retain the residual stresses [shown by finite-element simulations (Stefenelli *et al.*, 2013 ▸)], but thin enough to allow sufficient X-ray transmission, diffraction patterns can be collected by an area detector in transmission geometry while scanning the sample in the X-ray beam. The measurement of full Debye–Scherrer rings then allows texture to be measured (or at least estimated) from the azimuthal intensity distributions, and residual stresses to be determined from the distortion of the rings. This approach has since been used to measure important characteristics, including residual stress distribution, in a multitude of different coating systems such as TiN (Daniel *et al.*, 2014 ▸; Meindlhumer *et al.*, 2020*a* ▸), (Ti,Al)N (Riedl *et al.*, 2014 ▸; Zalesak *et al.*, 2016*a* ▸,*b* ▸; Bäcke *et al.*, 2023 ▸), TiAlON (Schalk *et al.*, 2019 ▸), CrN (Daniel *et al.*, 2013 ▸; Bartosik *et al.*, 2013 ▸; Daniel *et al.*, 2015 ▸), Cr–Al–C (Heinze *et al.*, 2023 ▸), TiW (Saghaeian *et al.*, 2019 ▸), TiB (Schalk *et al.*, 2014 ▸), Al_2_O_3_ (Tkadletz *et al.*, 2015 ▸), diamond (Gruber *et al.*, 2019 ▸; Hinzmann *et al.*, 2021 ▸) and different multilayers (Bartosik *et al.*, 2015 ▸; Keckes *et al.*, 2018 ▸; Tkadletz *et al.*, 2018 ▸; Jäger *et al.*, 2019 ▸; Klima *et al.*, 2019 ▸).

Shortly after its introduction, this approach [typically referred to as cross-sectional nano X-ray diffraction (CSnano­XRD)] was applied to map the residual stress fields in an indented multilayer CrN–Cr thin film (Stefenelli *et al.*, 2015 ▸) [and later also to study stress distribution and microstructural changes at scratch-tracks in a CrN–Cr bilayer (Meindlhumer *et al.*, 2020*b* ▸)]. However, as diffraction-based methods by definition only measure elastic strains, *ex situ* stress mapping cannot reveal information about the actual stress and strain distribution during loading. Later, a sample environment specifically developed for *in situ* characterization during nanoindentation using CSnanoXRD was therefore used to provide unique insights into the stress distribution in a bilayer TiN coating below a diamond wedge tip (Zeilinger *et al.*, 2016 ▸). The geometry, with a wedge longer than the thickness of the indented lamella, produced a close-to-constant stress state through the lamella thickness (along the X-ray beam direction), suitable for mapping with the CSnanoXRD geometry previously developed. Approximately at the same time, *in situ* scattering was used to characterize strain fields during indentation of a Zr-based metallic glass using a scanning probe approach (Gamcová *et al.*, 2016 ▸). However, a pyramidal Berkovich tip was used to indent a 40 µm-wide lamella, and, thus, the strain state was not homogeneous through the thickness. Subsequently, other studies using the CSnanoXRD approach have been used to resolve stress field evolution in multilayered CrN coatings (Ecker *et al.*, 2020 ▸) and CrN–AlN superlattice thin films (Todt *et al.*, 2020 ▸) during indentation. Todt *et al.* (2021 ▸) used *in situ* CSnanoXRD to investigate the effects of residual stress gradients and indenter geometry on stress field development during nanoindentation, whereas Meindlhumer *et al.* (2021 ▸) used clamped microcantilevers to investigate the evolution of stress fields during crack growth and arrest in CrN–Cr multilayers. Importantly, it was shown that the intensity at small angles (close to the beam stop) could be used to image cracks, allowing identification of their initiation and propagation (Meindlhumer *et al.*, 2021 ▸). Recently, Zauner *et al.* (2022 ▸) demonstrated that the stress state in a notched microcantilever from a CrN coating could be mapped during application of fatigue cycles. These advances show that *in situ* CSnanoXRD during nanomechanical testing has great potential as a tool for interrogating the deformation and fracture mechanisms in advanced coating systems during complex loading situations. Consequently, access to suitable sample environments and hard X-ray nanoprobes is critical in order to allow such experiments to be performed.

In this report, we describe a sample environment for *in situ* nano- and micro-mechanical testing recently commissioned at the nanodiffraction endstation (Carbone *et al.*, 2022 ▸) on the NanoMAX beamline (Johansson *et al.*, 2021 ▸; Björling *et al.*, 2020 ▸) located at the 3 GeV ring of the MAX IV synchrotron, Lund, Sweden. The beam is focused by a fixed-curvature Kirkpatrick–Baez (KB) mirror system, which creates a diffraction-limited focus of 200–40 nm in the energy range 5–28 keV. The long focal length of the KB mirror system (the mirror centre to focal point distance is 310 mm for the horizontal focusing and 180 mm for the vertical focusing) provides sufficient space (around 80 mm in the beam direction) for large sample environments like the presented nanoindenter. After characterizing the response of the nanoindenter, we perform diffraction mapping of the strain and stress fields in thin films during indentation in order to demonstrate its capabilities. Monitoring the azimuthal intensity distribution also allowed qualitative mapping of the plastic deformation, and using the scattered signal in the vicinity of the beamstop allows visualization of interfaces and cracks formed during deformation.

## 2. Experimental setup

### 2.1. Sample environment

The implemented sample environment comprises a commercial nanoindenter (Alemnis Standard Assembly) supplied by Alemnis, Thun, Switzerland. The indenter tip is mounted on a piezo-actuated displacement head equipped with an integrated displacement sensor for closed-loop operation (maximum displacement 40 µm, specified displacement resolution <1 nm). At the time of the commissioning experiments presented here, a 0.5 N load cell, with a specified RMS noise level around 4 µN, was used. Currently, an additional 1.5 N load cell (8 µN RMS noise) and grippers for microtensile testing are also available. Load cells with even higher maximum loads, up to 4 N, are available and retrofittable. A piezoelectric three-axis micro-positioning system is used for sample positioning and tip approach (26 mm lateral range in all three directions), with integrated position sensors for closed-loop operation (<2 nm specified resolution). The specific indenter was chosen for two reasons: (i) the true displacement control, which is important for controlled crack extension and fracture testing; and (ii) its modular architecture, which allows for an extension of the testing capabilities by retrofittable add-ons, *e.g.* high-temperature testing (up to 1000°C in vacuum or 200°C in air), low-temperature testing (−150°C), ultra-high-strain-rate testing, scratch testing, tensile testing (using microgrippers), electrical testing and liquid cells. Multiple tests are realizable depending on the chosen configuration and tip geometry, including nanoindentation, micropillar compression and microcantilever bending. The access to a wide range of retrofittable add-ons is an important point, as it allows continuous development of the capabilities of the sample environment in order to meet the demands of the research community. The use of a commercial *in situ* indenter designed for use in scanning electron microscopes furthermore enables pre-characterization of the sample and setup, promoting optimal use of beam time.

Two methods are available to control the nanoindenter. The Alemnis Micro Indenter Control Software (AMICS) offers a graphical user interface (GUI). Additionally, the nano­indenter standard commands are available as ASCII commands following the SCPI standard via the command line. The communication between software and hardware is realized via a socket interface. On top of it, a TANGO device server written in Python was realized, allowing the full integration of the nanoindenter into the beamline control system. A parallel operation of the AMICS GUI and the remote control at the beamline is possible.

**Figure 1:**
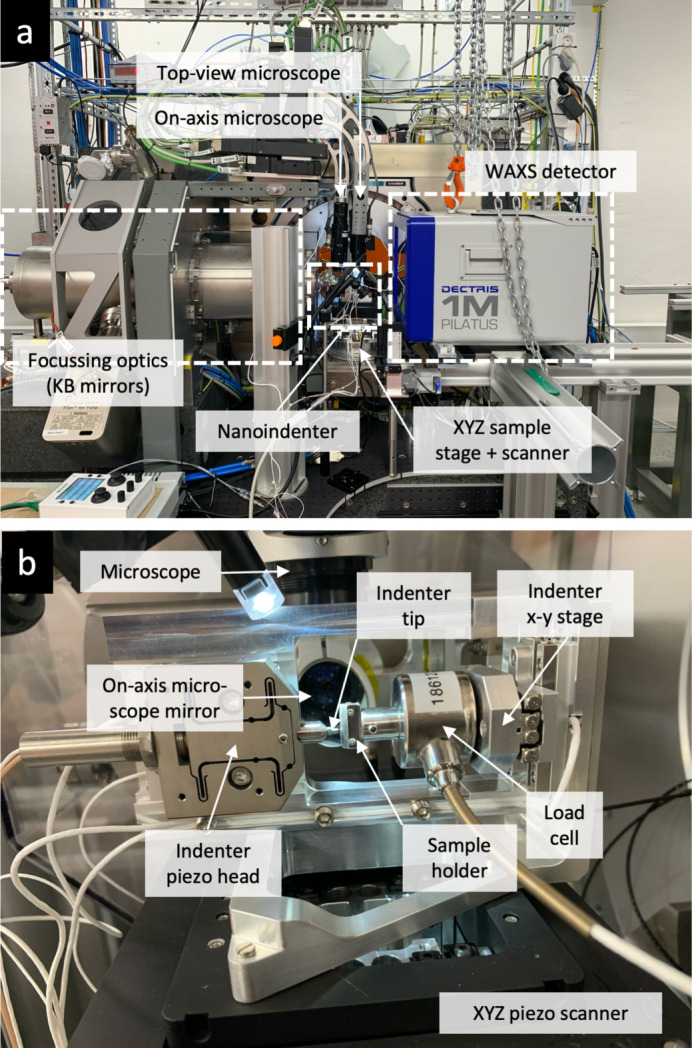
(*a*) Photograph of the sample environment installed at the diffraction endstation at NanoMAX. (*b*) Close-up of the indenter as viewed from the detector side.

Figure 1 ▸(*a*) shows an overview of the sample environment on top of the *xyz* sample stage and scanner, between the downstream detector and the upstream KB mirror vacuum chamber. Notably, the two long-distance optical microscopes allowing visual access both from the top and the on-axis direction were used for coarse positioning of the sample relative to the tip. The nanoindenter was mounted in horizontal mode, standing on the side of the frame [see Fig. 1 ▸(*b*)] to allow visual access for the top-view microscope. Custom-made in-house sample holders were designed to clamp the samples, as will be described later.

### 2.2. *In situ* indentation experiments

#### 2.2.1. Material and sample preparation

**Figure 2:**
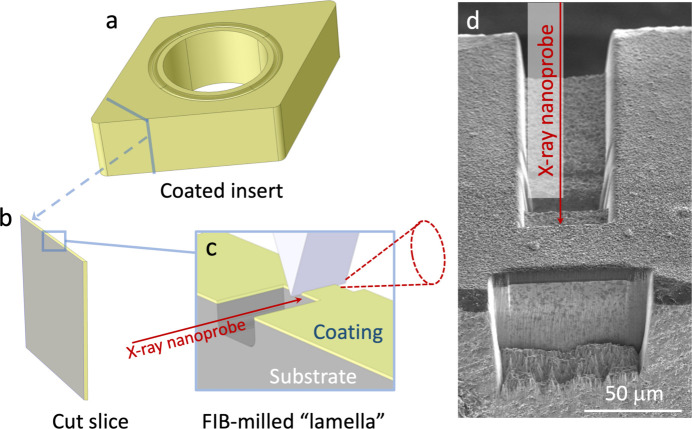
Schematic illustration of the sample preparation. (*a*) Coated insert, from which an approximately 300 µm-thick slice is cut (*b*). (*c*) Focused ion beam (FIB) milling is used to locally reduce the thickness of the slice to produce a lamella with a thickness below 50 µm (50 µm is the width of the indenter wedge). The lamella is placed close to the downstream edge in order to avoid shadowing of the detector and allow the full 111 diffraction ring to be captured. (*d*) SEM image of the FIB milled trench, viewed from the downstream side.

To directly demonstrate the method’s usefulness for industrially relevant materials we selected coarse-grained textured coatings deposited by chemical vapour deposition (CVD). These coatings represent commercial-grade cutting tool inserts for metal machining applications. Two different bilayer coating systems were used: 4 µm Ti_0.2_Al_0.8_N on top of a 1 µm TiN layer, and 7 µm Al_2_O_3_ on top of TiCN of similar thickness. Both coatings were deposited on WC–Co cutting tool inserts, and more details on deposition conditions and microstructures are given by Qiu *et al.* (2021 ▸) [(Ti,Al)N/TiN] and Shoja *et al.* (2020 ▸) (Al_2_O_3_/TiCN). Slices with approximate thicknesses of 300 µm were cut from the insert by a low-speed diamond saw, Figs. 2 ▸(*a*) and 2(*b*). Areas with dimensions (width × height) of around 50 µm × 12 µm [(Ti,Al)N/TiN] or 50 µm × 20 µm (Al_2_O_3_/TiCN) were locally thinned down to a ‘lamella’ with a thickness of around 40 µm using focused ion beam (FIB) milling in an FEI Versa 3D focused ion beam-scanning electron microscope (FIB-SEM), Fig. 2 ▸(*c*). The lamella is placed close to the downstream edge of the sample in order to avoid shadowing of the diffraction signal by the surrounding bulk material and allow the full 111 diffraction ring to be captured, Fig. 2 ▸(*d*).

#### 2.2.2. Nano-diffraction mapping

**Figure 3:**
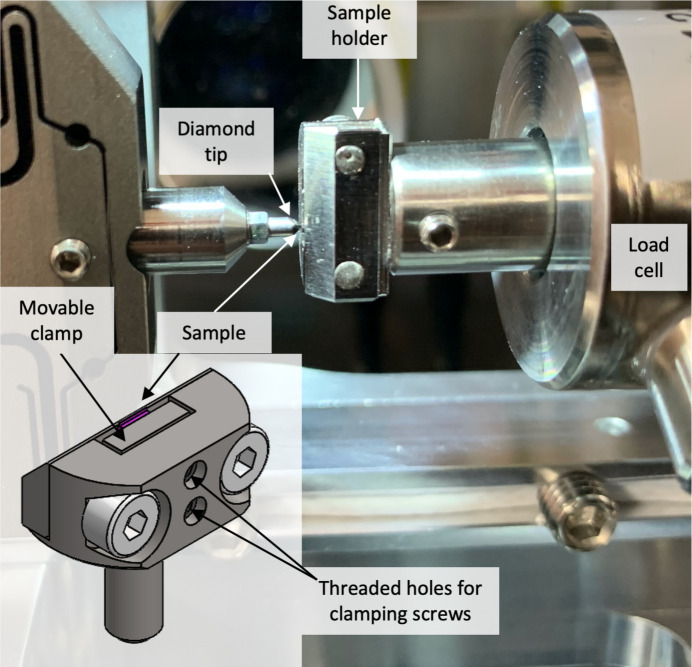
Photograph of the sample holder, as seen from the downstream side, mounted on the indenter. The insert shows a schematic of the holder.

The samples were mounted in a specifically designed sample holder, shown in Fig. 3 ▸. Care was taken to design holders which allow easy mounting with good inherent alignment. The depth of the slot where the slice was inserted was chosen so that the sample protrudes by approximately 200–300 µm above the top surface of the holder when the lower edge of the sample is fully supported by the bottom of the slot. Supporting the sample at the bottom ensures that no sliding occurs and that the top surface of the sample is perpendicular to the *z*-direction (see Fig. 5 for the definition of the coordinate system). The movable clamp was fixed with two screws to ensure tight contact along the entire slice. The downstream edge of the sample holder was chamfered at 45° to avoid shadowing of the diffraction signal.

**Figure 4:**
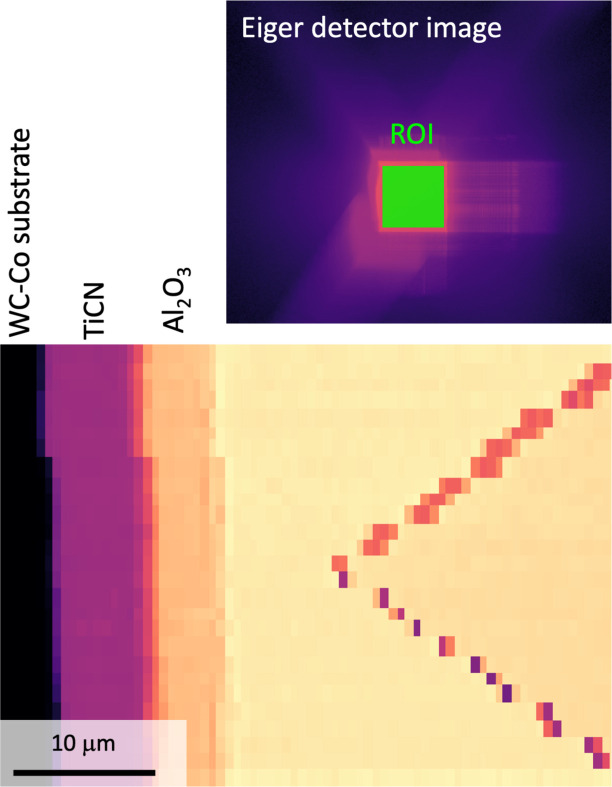
Part of a far-field Eiger2 X 4M detector image and the diamond wedge and bilayer coating visualized in dark-field contrast using the indicated region of interest (ROI) during fine positioning of the tip. The particular scan was performed using fly scanning with a step size 0.5 µm in the horizontal direction (fast motor) and 1 µm in the vertical direction.

**Figure 5:**
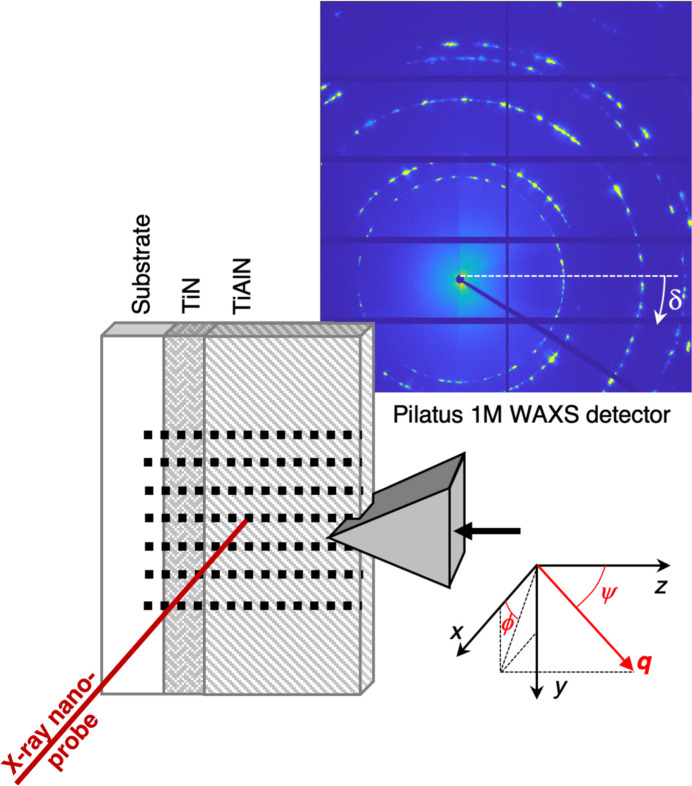
Geometry of the stress mapping experiments. Note that the sample coordinate system (which is different from the beamline coordinate system) was chosen so that the *z*-axis coincides with the out-of-plane direction of the coating.

The coating in the remaining 40 µm-wide lamella was indented from the top using a 50 µm-long diamond wedge (slightly wider than the lamella width) with an opening angle of 70° from Synton-MDP AG, Nidau, Switzerland, aligned with the X-ray beam [Fig. 2 ▸(*c*)]. The sample was first moved using the indenter sample stage to place the lamella directly below the wedge tip using the two optical microscopes. The entire indenter was then moved using the stage motors to bring the diamond tip into the X-ray focus. Importantly, the use of a polished diamond tip allowed visual access to the top surface of the lamella using the top microscope through image reflection on the edge of the wedge. This enabled very accurate positioning of the tip in the *x*-direction (see Fig. 5), which could later be fine-tuned by ensuring that the lamella was in the focal plane of the on-axis microscope which in turn was calibrated to coincide with the X-ray focal plane at the highest magnification. Using the optical microscopes, the tip was moved as close as possible to the top of the coating. The limited resolution of the long-working-distance optical microscopes required fine positioning by performing scans of the region containing the tip and lamella using either a downstream far-field detector (Eiger2 X 4M) placed in a vacuum flight tube at approximately 4.5 m from the sample [see Fig. 4 ▸ for an example of a map of the diamond wedge (edge-on) positioned about 7 µm above the Al_2_O_3_/TiCN coating] or a combination of total scattered intensity and streaking from the diamond faces on the detector used for collection of wide-angle scattering (a Pilatus 1M, hereafter denoted as the WAXS detector) placed between 136 mm and 196 mm downstream (different for different measurements based on material and photon energy to ensure at least one full Debye–Scherrer ring on the detector), see examples in Fig. 13. To access the far-field detector, the WAXS detector was manually moved out of the beam path, but the kinematic mounts ensured accurate repositioning. Once in the correct position at the centre of the wedge, the tip was brought into brief contact using the ‘auto-approach’ feature of the indenter control software and then retracted a few micrometres before the first scan was performed. Diffraction mapping of an area (*y* × *z*, defined in the sample coordinate system shown in Fig. 5 ▸) of approximately 5 µm × 7 µm [(Ti,Al)N] or 7 µm × 7 µm (Al_2_O_3_) was performed with a step size Δ*y* = Δ*z* = 100 nm (see Fig. 5 ▸).

Diffraction patterns were recorded in transmission geometry using the WAXS detector. Calibration was performed using *PyFAI* version 0.21 (Kieffer *et al.*, 2020 ▸) using diffraction patterns obtained from multiple integrated frames from NIST standard Si or LaB_6_. The photon energy and probe size were 16 keV and 60 nm (FWHM) for (Ti,Al)N and 14 keV and 70 nm (FWHM) for Al_2_O_3_ (Björling *et al.*, 2020 ▸). An acquisition time of 1 s per point was used, which, with overhead for stage movement, yielded a total time of around 1.5–2 h per map. Other scanning strategies, such as fly scanning, can significantly reduce this time, which has been verified in separate experiments not included in this study.

During the acquisition of each map, the applied load was held constant (force control mode). Maps were acquired before and after indentation, as well as *in situ* at different load levels. The logging frequency of the indenter (recording force and displacement) was set to between 2 Hz and 10 Hz. No post-indentation compliance correction was applied to the recorded displacement data, as the system compliance with a tailor-made sample holder and sample with unique geometry and uncertainty in alignment was not known.

## 3. Results

### 3.1. Setup response

**Figure 6:**
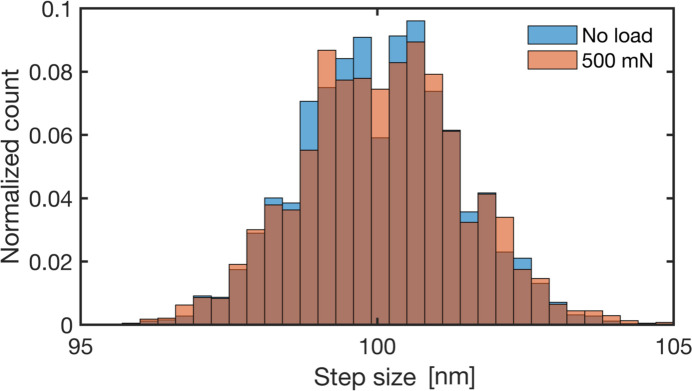
Measured step size Δ*z* of the fast-scanning motor with and without the load applied by the indenter. The nominal step size was 100 nm.

Accuracy in the response of the piezo scanning stage is critical for sub-100 nm probes, especially with the relatively heavy sample environment (total weight of the nanoindenter system is around 700 g) mounted on top of it. Figure 6 ▸ shows histograms of the measured step size, Δ*z*, from the scans before loading and with an applied load of 500 mN. The mean is within 0.1% of the nominal value (100 nm), and the distribution is symmetric with a very small standard deviation (1.3 nm), implying that scanning with step sizes matching the beam size, or even smaller, is feasible.

**Figure 7:**
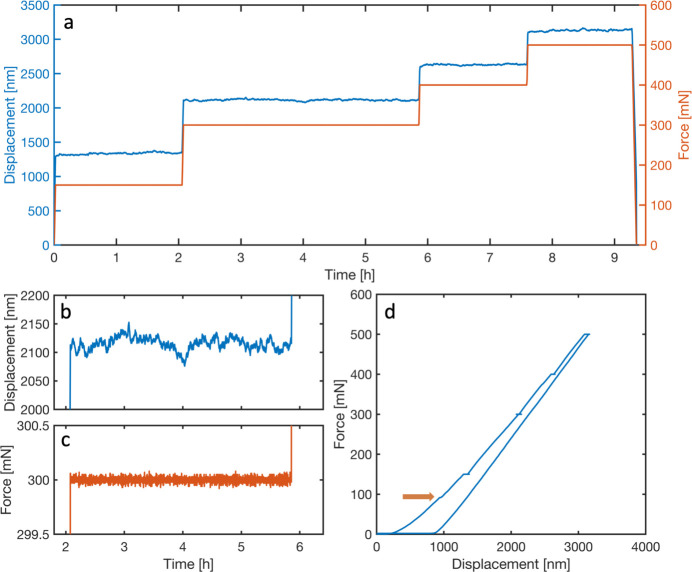
Displacement and force response of the nanoindenter recorded during the experiment. (*a*) Displacement and force versus time. (*b*, *c*) Close-ups of the displacement and force responses, respectively, during the hold period at 300 mN. (*d*) Raw (not corrected for system compliance) force–displacement curve. The arrow in (*d*) indicates a small event which is potentially related to crack initiation.

The response of the nanoindenter during the scans with load applied is shown in Fig. 7 ▸, and important characteristics are given in Table 1 ▸. Due to the force control mode of operation, the measured force drift is essentially zero, and the force noise is of the order of 7 µN. The displacement signal, on the other hand, undergoes measurable drift during the hold periods. The origin of the displacement drift can be both actual creep/relaxation effects in the sample or due to creep in the piezo actuators. The total drift during the mappings lies in the range 4–40 nm. The displacement noise, when correcting for the average drift, is of the order of 10 nm, but as the displacement signal undergoes slower changes this is an overestimation of the actual noise level. The slower changes are likely due to temperature variations in the experimental hutch. The nanoindenter controller is placed relatively close to the sample position to keep signal cables as short as possible and reduce electronic noise. The generated heat of the controller electronics is intermittently ejected from the casing by fans, affecting the thermal stability of the nanofocusing setup and the nanoindenter mechanics. Future experiments will include ‘shielding’ the sample position from the exhaust air directing the airflow immediately towards the air outlets in the hutch, and ultimately placement of the controller in a ventilated cabinet. Encouragingly, no obvious signs of the motor stepping in either force or displacement response could be observed.

The measured response of the indenter indicates that it performs according to requirements, enabling mapping with spatial resolution approaching the beam size, as long as the total hold time remains reasonable. Minimizing the total test time is the standard recommendation in nanomechanical testing procedures in order to mitigate drift-related issues.

### 3.2. Strain and stress mapping of Ti_0.8_Al_0.2_N

A framework for analysis of the stress field during nanoindentation of thin films with the current geometry was developed by Zeilinger *et al.* (2016 ▸). Although based on several simplifying assumptions, the approach was shown to give good qualitative agreement with finite-element simulations. Here, we follow the methodology outlined by Zeilinger *et al.* (2016 ▸), briefly described below, to map the strains and stresses in the (Ti,Al)N coating in order to demonstrate the method and sample environment. We also note that, for a full quantitative analysis, a more complex approach applying ψ-dependent stress factors *F*_*ij*_(ψ, *hkl*) accounting for texture, direction-dependent elastic interactions and grain morphology is required (Welzel *et al.*, 2005 ▸). However, for the current purpose (demonstration of the sample environment capabilities) the simplified analysis is sufficient.

**Figure 8:**
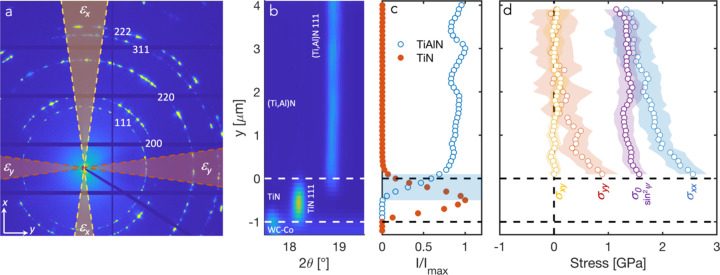
(*a*) Single detector frame from the undeformed coating. The shaded regions on the detector image correspond to the sectors used for evaluation of the in-plane (ε_*y*_) and out-of-plane (ε_*z*_ strains). (*b*) Diffractograms (intensity versus 2θ) as a function of position in the coating before application of load, showing (from bottom to top) WC–Co substrate, TiN bonding layer and (Ti,Al)N coating. (*c*) Normalized intensity distribution of the TiN and (Ti,Al)N 111 peaks with position in the coating. (*d*) Residual stresses obtained from fitting of equation (6), see text for more description. Also included is the in-plane stress calculated by the 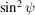 method, showing good agreement towards the top but deviations towards the (Ti,Al)N/TiN interface.

Figure 8 ▸(*a*) shows a typical single detector image obtained from the undeformed coating. As expected from the large grain size relative to the beam dimensions, the rings are spotty, and the intermittent azimuthal distribution shows the deposition-induced texture. To obtain the azimuthal strain distribution required for strain and stress evaluations each pattern was divided into 36 sectors (10° cakes) along the azimuthal angle (δ, see Fig. 5 ▸), which were individually reduced. For each sector the (Ti,Al)N 111 peak was fitted with a pseudo-Voigt peak shape function using the *LIPRAS* Matlab interface (version 1.466.2.0) (Esteves *et al.*, 2017 ▸), allowing calculation of the diffraction strain at each each position (*y*, *z*) from the 111 *d*-spacing, 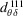 , according to 

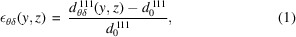

where θ is half the diffraction angle and 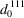 is the strain-free *d*-spacing. For an equibiaxial stress state 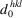 can be found when the diffraction vector satisfies ψ^*hkl*^ = 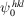 , where 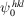 can be calculated from the plane-specific diffraction elastic constants (DECs) 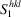 and 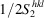 . Unfortunately, DEC values are not available for the present coating, and the presence of both crystallographic texture and grain shape anisotropy significantly complicates the analysis, as mentioned above. For method demonstration, we therefore use the values obtained from the Kröner model (Kröner, 1958 ▸), as calculated by the *IsoDEC* software (Gnäupel-Herold, 2012 ▸) using single-crystal elastic constants for Ti_0.2_Al_0.8_N (Tasnádi *et al.*, 2010 ▸) ( 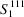 = −0.3 × 10^−3^ GPa^−1^ and 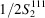 = 2.3 × 10^−3^ GPa^−1^). However, as the accuracy of the strain-free lattice parameter is critical for the strain (and hence subsequent stress) calculations, the estimation of *d*_0_ from 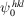 obtained under the above assumptions was not deemed suitable. Instead, 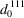 was found by an iterative procedure where the stresses found from the least-squares-fitting procedure described below yielded zero stress in the out-of-plane direction (σ_*zz*_ = 0) at the sample surface.

In the following stress analysis, we adopt the same assumptions as outlined by Zeilinger *et al.* (2016 ▸): the residual stress state in the as-deposited coating was approximated as triaxial with non-zero components σ_*ii*_ ≠ 0 and σ_*yz*_ ≠ 0 and in-plane equibiaxial stress, σ_*xx*_ = σ_*yy*_ = σ_0_. The remaining shear stress components were assumed to be negligible, σ_*xy*_ = σ_*xz*_ = 0. While it is possible to argue that other assumptions would be more suitable (*e.g.* a fully equibiaxial stress state where also σ_*zz*_ = σ_*yz*_ = 0, or full or partial relaxation of in-plane stresses due to sample preparation so that σ_*xx*_ ≠ σ_*yy*_), the main purpose of this study is to demonstrate that the data allow reliable extraction of the stresses and we therefore follow Zeilinger *et al.*, in order to facilitate a direct comparison. Under the above assumptions, the relationship between the stresses and the measured strains [expressed in (ψ, ϕ) space] is given by 

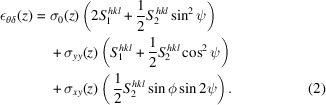

Using the following relationships,

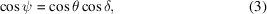




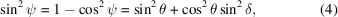




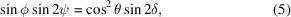

we obtain the following expression for ε_θδ_, 

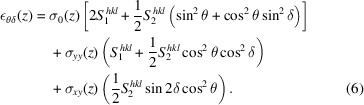

Note that equation (6) assumes no lateral variations of the stresses, and all detector images recorded at the same height (*z*) were therefore averaged before reduction and fitting to increase the statistics.

Figure 8 ▸(*b*) shows a surface plot of the azimuthally integrated intensity versus diffraction angle for a selected 2θ range as a function of position in the coating. At the bottom, the upper part of the WC–Co substrate is seen, on top of which the TiN bonding layer is visible. As seen from the normalized intensity versus *z* profiles for the (Ti,Al)N 111 and TiN 111 peaks in Fig. 8 ▸(*c*), the overlap of the signals from the two layers is of the order of 500 nm. In the following, we define *z* = 0 as the position where the TiN intensity has vanished, in order to avoid complications from the overlap region. In the case of an ideally smooth interface perfectly aligned with the beam, we would expect an overlap region corresponding to the beam size (60 nm). The interface roughness of the specific coatings investigated here was relatively small, of the order of 100 nm, which would give an overlap (contribution from both beam size and roughness) of 200 nm. Further effects arise from the interface not being completely parallel to the X-ray beam. Considering the thickness of the lamella of 40 µm, an overlap of 500 nm (accounting for the beam size contribution) corresponds to a misalignment below 1°. Further possibilities to improve the alignment of the interface with the beam through optimized sample mounting or sub-stages for isolated sample tilting should be explored in order to approach the ultimate spatial resolution defined by the beam size. In particular, this will be important for the investigation of multilayer coatings.

The iterative least-squares fitting of equation (6) to ε_θδ_(*z*) resulted in 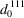 = 2.350 (7) Å [corresponding to a strain-free lattice constant *a*_0_ = 4.071 (5) Å], and the resulting residual stress profiles are plotted in Fig. 8 ▸(*c*). The in-plane stress, σ_*yy*_, is of the order of 2.5 GPa at the (Ti,Al)N/TiN interface, and decreases to around 1.5 GPa towards the surface. This agrees well with previous reports of residual tensile stress of 1.45 GPa in as-deposited coatings (Tkadletz *et al.*, 2020 ▸). The out-of-plane stress, σ_*zz*_, is zero at the surface (as a result of the iterative fitting) while becoming tensile in the order of 1 GPa towards the (Ti,Al)N/TiN interface. This is similar to the reports by Zeilinger *et al.* (2016 ▸), who found non-zero out-of-plane stresses at the interface between the upper and lower parts of a CVD TiN coating, where the temperature had changed half-way through the deposition. They attributed this to the constraining effect of the upper part of the coating on the ability of the lower part to relax stresses during the deposition. In our case, the location of the non-zero out-of-plane component is confined to the lower half, increasing towards the (Ti,Al)N/TiN interface, and the same argument could be made. However, we note that there may be other explanations, such as possible gradients in chemistry, grain size/morphology and texture. Previous studies (Qiu *et al.*, 2020 ▸) have shown that the Ti:Al ratio is constant through the thickness, except for the innermost 200–300 nm, which is too small to explain the present gradient in σ_*zz*_. On the other hand, gradients in texture and grain morphology extend further into the coating (Qiu *et al.*, 2020 ▸), and as the (Ti,Al)N is elastically anisotropic this could potentially affect the DECs, and consequently the results of the linear regression where these values have been assumed to be constant throughout the coating. The shear stress, σ_*xz*_, is zero throughout the thickness, as expected.

Furthermore, the collection of data over the full azimuthal range allows us to compare the use of equation (6) and the standard assumption of linear *d* versus 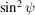 response when determining the residual stresses. Under the assumption of an equibiaxial stress state, the in-plane stress can be found from a linear fit of 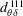 versus 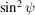 at each position (*y*, *z*),

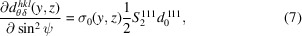

where 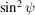 corresponding to each (θ, δ) combination is calculated using equation (4). As seen in Fig. 8 ▸(*d*), the 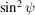 approach provides very similar values in the outer part of the coating (where the equibiaxial stress state assumption is valid) but deviates in the lower part. Again, we must remember that the here-neglected complicating effects of texture, grain morphology and direction-dependent elastic interactions will also induce non-linearity in the *d* versus 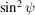 response not associated with the stress state [see *e.g.* Welzel *et al.* (2005 ▸)].

**Figure 9:**
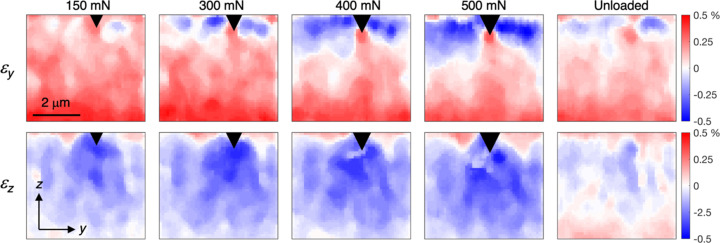
Evolution of the strain fields (ε_*y*_ and ε_*z*_) during indentation and after unloading. The black triangle indicates the approximate position of the indenter tip.

Figure 9 ▸ shows the maps in-plane (ε_*y*_) and out-of-plane (ε_*z*_) elastic strains (smoothed using a median filter with a 5 × 5 neighbourhood) during progressively increasing indentation load, as well as after complete removal of the load. Note that the top of the map does not exactly correspond to the free upper surface of the coating. The uppermost part was excluded since the surface is ‘smeared’ by surface roughness and slight misalignment, as discussed above. The strain values were obtained by averaging the strains in the ±10° sectors around the 0 and 180° and 90 and 270° positions on the detector, as shown in Fig. 8 ▸(*a*). The resulting strain fields are in good agreement with expectations. In-plane compressive strains (ε_*y*_) develop at the side of the diamond tip, whereas tensile strains develop underneath due to the cleaving effect of the sharp wedge. The out-of-plane strains (ε_*z*_) show the inverse behaviour. Small residual strains remain after unloading.

**Figure 10:**
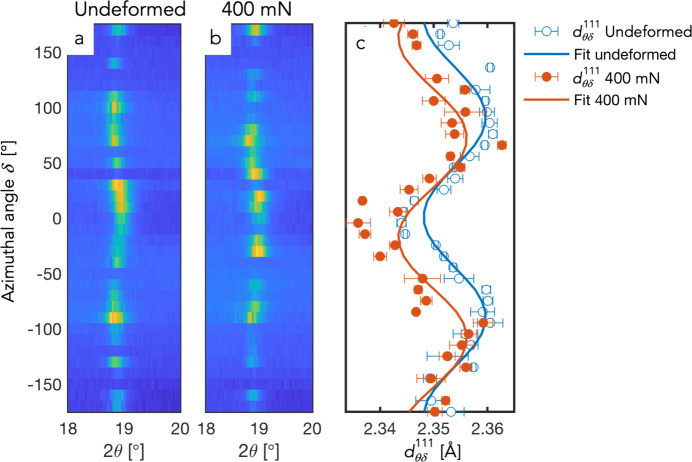
(*a*, *b*) Azimuthal intensity distribution around the 111 diffraction angle from single frames taken from undeformed sample and during indentation with 400 mN load, respectively. (*c*) Fitted 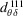 -spacings and corresponding fits of equations (6) and (8) for extraction of stresses [same frames as in (*a*) and (*b*)].

Following Zeilinger *et al.* (2016 ▸), σ_*xx*_ was assumed to be unaffected during indentation, fixed to the previously determined value of σ_0_. This assumption is necessary due to low sensitivity to strains in the *x*-direction, but also reasonable since this is the direction aligned with the diamond wedge. A benefit of the low sensitivity is that the exact value of σ_0_ is not critical, as long as it is reasonable. The stress–strain relationships are consequently given by

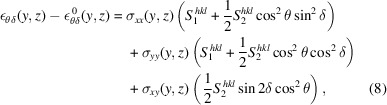

where 

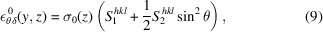

and σ_0_(*z*) is the average in-plane residual stress at *y* [as previously determined, see Fig. 8 ▸(*d*)]. From the above assumptions, the remaining non-zero stress components can then be found from weighted least-squares fitting of equation (8) at each position (*y*, *z*), where the weight corresponded to the inverse of the squared *d*-spacing error (*w*_*i*_ = 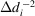 ). All fitting and evaluation were performed by in-house MATLAB scripts. Typical fitting results are shown in Fig. 10 ▸. Examples of the azimuthal distribution of the intensity around 2θ ≃ 19° (the 111 reflection) are shown in Figs. 10 ▸(*a*) and 10(*b*), where the stress-induced ellipticity can be seen. The corresponding 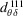 -spacings for the two frames in Figs. 10 ▸(*a*) and 10(*b*) are shown in Fig. 10 ▸(*c*), together with the results from the weighted linear least-square fits of equations (6) and (8), respectively.

**Figure 11:**
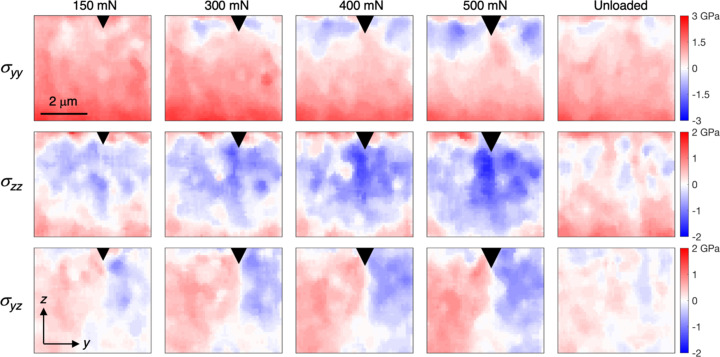
Evolution of the stress fields (σ_*yy*_, σ_*zz*_ and σ_*yz*_) in (Ti,Al)N during indentation and after unloading.

The evolving stress fields are shown in Fig. 11 ▸ (same regions as in Fig. 9 ▸, smoothed using a median filter with a 5 × 5 neighbourhood). Zones of very high compressive stresses in the in-plane direction (σ_*yy*_) develop at the sides of the wedge, as expected. In the out-of-plane direction a growing region with increasingly compressive stresses (σ_*zz*_) can be seen. An anti-symmetric shear stress field (σ_*yz*_) progressively develops with increasing load. As expected, most of the indentation-induced stresses are removed as the sample is unloaded, emphasizing the importance of *in situ* measurements rather than post-test stress mapping for understanding the behaviour. These results are in general agreement with previous reports of stress field development during indentation of bilayer TiN (Zeilinger *et al.*, 2016 ▸), trilayer CrN (Ecker *et al.*, 2020 ▸) and multilayer CrN–AlN (Todt *et al.*, 2020 ▸) coatings synthesized by physical vapour deposition. While these coatings had more complex multilayer structures, the small grain size and close to random crystallographic texture enable more accurate stress evaluation, consequently serving as good benchmarks. We note that the magnitude of the in-plane tensile stress decreases with increasing load, which is surprising since the cleaving effect is expected to increase. Similar trends were reported for the multilayer coating (Todt *et al.*, 2020 ▸), and attributed to the early formation of cracks which allowed relaxation of the stresses. Indeed, a crack was observed when the sample was imaged in an SEM after unloading. Additionally, the force-displacement curve [marked by the arrow in Fig. 7 ▸(*d*)] shows a small event at slightly below 100 mN, which is indicative of cracking. It is thus likely that the coating cracked before reaching a load of 150 mN. The growth of the crack explains the gradual relaxation of the in-plane stresses.

As a final note, we also investigated the effect of assuming a constant value of σ_0_ (= 1.5 GPa, as obtained from the 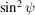 analysis) in equation (9) on the resulting stress fields during indentation, and concluded that this did not significantly affect the results.

### 3.3. Deformation mapping and crack detection in Al_2_O_3_/TiCN

**Figure 12:**
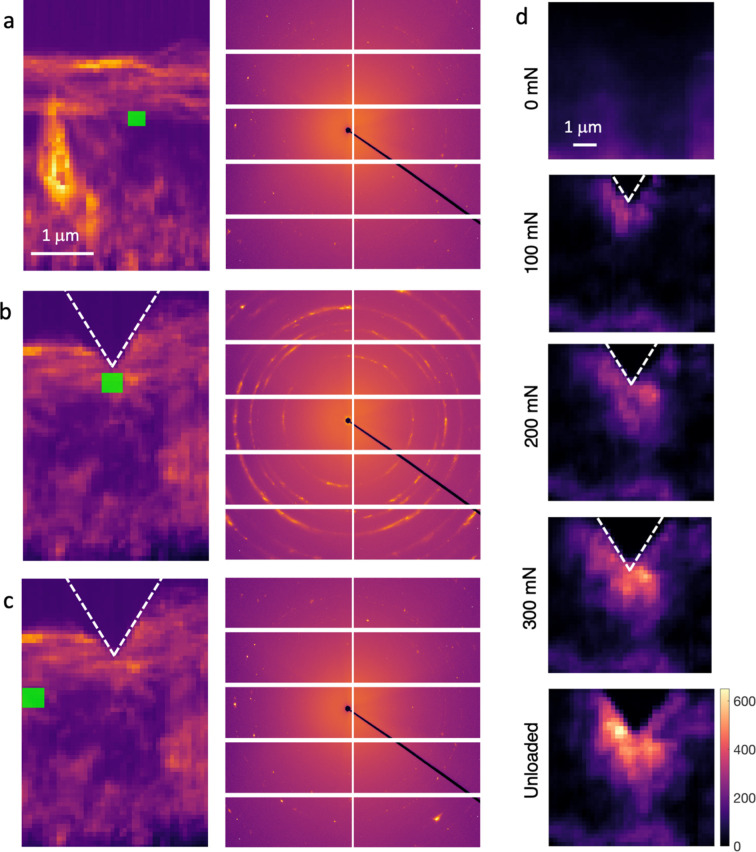
(*a*)–(*c*) Change in diffraction pattern due to induced plastic deformation. Each subfigure shows a map of the integrated total intensity at each position in the coating on the left-hand side, and the summed detector images from the ROI indicated by a green rectangle on the right-hand side. The summed detector images in (*a*) and (*b*) correspond to approximately the same position before, (*a*), and during, (*b*), indentation. As a reference, panel (*c*) shows the detector images at a position away from the indent in the probed coating. Note that the individual spots corresponding to Al_2_O_3_ are difficult to see in (*a*) and (*c*), whereas the smeared rings due to plastic deformation are clearly visible in (*b*). (*d*) Deformation mapping during indentation. The top image shows the total number of pixels in 2θ ranges corresponding to selected Bragg peaks (012, 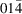 , 110 and 113) with an intensity above the noise threshold before indentation. The following images show the increase in the number of such pixels (the scale bar at the bottom image is valid for all maps).

The Al_2_O_3_/TiCN bilayer offered a more challenging structure to map due to the very limited interaction between Al_2_O_3_ and the X-ray beam combined with large grain size. It nevertheless offered a possibility to demonstrate the other capabilities of *in situ* nanoindentation, *i.e.* deformation mapping and crack visualization. Comparing the summed detector images of a smaller region of interest (ROI) we note an evolution of the diffraction patterns recorded in the vicinity of the indenter tip. Figure 12 ▸(*a*) shows the rings in the as-deposited sample, which are initially very spotty. Only a few very sharp Bragg peaks can be seen and the rings are hardly visible on the detector. The spots become broad arcs below the indenter tip, Fig. 12 ▸(*b*), which indicates large plastic deformations creating grains with wide orientation distributions. Further away from the indenter tip, the spotty rings are retained. As the ‘smearing’ of the initially sharp Bragg peaks redistributes the intensity over a larger number of pixels on the detector, plasticity can be monitored by counting the number of pixels with intensity above a selected noise threshold. At each position, the total number of pixels with an intensity of a threshold (20 counts was used to exclude noise) within ±0.15° of the nominal 2θ position corresponding to the 012, 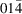 , 110 and 113 reflections were counted. The result is seen in Fig. 12 ▸(*d*), showing the average number of counted pixels from the different reflections for the as-deposited coating, and the increase in the number of counted pixels during indentation. The plastic zone develops as the load increases and remains after unloading. Furthermore, by stepping through the maps and inspecting the detector images corresponding to individual positions it is possible to identify arcs originating from single grains. As each grain is probed at multiple positions (due to the small step size relative to the grain size) it is possible to follow the orientation at different positions in the grain. This possibility is not further explored here, but could open very exciting possibilities for detailed characterization of plasticity. This is particularly true if combined with sample rotation to obtain reciprocal space maps, or an increased energy bandwidth.

Finally, we note that there is a wealth of information in the scattered signal intensity in the vicinity of the beam stop. This was exemplified by Todt *et al.* (2020 ▸) where the small-angle scattering (SAS) signal was measured at the P03 beamline at PETRA III (DESY, Hamburg, Germany) by moving the area detector from the WAXS positions (198 mm downstream of the sample) to a second position at a distance of 928 mm. This experimental configuration allowed for mapping the multilayer periodicity and rotation during the indentation of a CrN–AlN superlattice film. We have also previously demonstrated that the periodicity in nanolamellar coatings can be determined from the SAS signal measured by a WAXS detector placed at a distance similar to the present study, both at NanoMAX and P03 (Bäcke *et al.*, 2023 ▸). Meindlhumer *et al.* (2021 ▸) used the scattered signal around the beamstop to identify the initiation of a crack at the root of a pre-machined notch during the bending of a multilayer CrN–Cr microcantilever.

**Figure 13:**
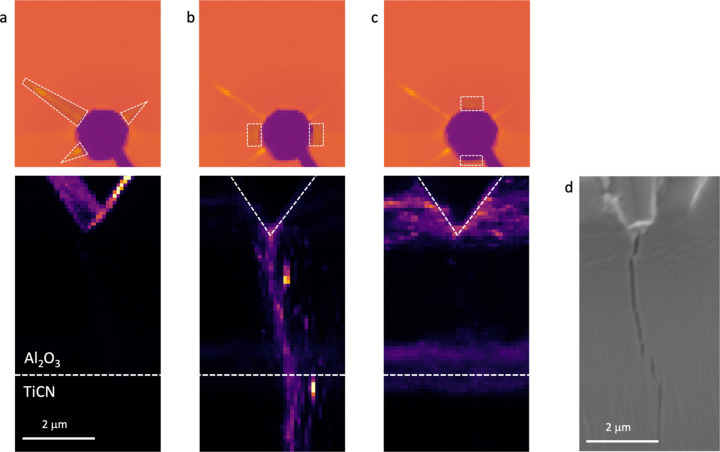
Visualization of the diamond tip and an indentation-induced crack using the scattered signal in the vicinity of the beamstop. (*a*) Diamond tip (bottom) visualized as the positions from which the streaks indicated by dashed regions (top) originate. Using the intensity from positions indicated in (*b*) (top), the crack can be clearly seen (bottom), whereas there is no indication of the crack when using the scattering signal as indicated in (*c*). The horizontal dashed line corresponds to the approximate position of the Al_2_O_3_/TiCN interface. (*d*) SEM image of the sample after testing, showing a single crack with the same shape as that visualized by SAS. Note that the sample was tilted in the SEM and the scale (particularly in the vertical direction) is distorted.

Here we use the directional-dependent signal at small scattering angles to image both the indenter tip during deformation and cracks and interfaces in the coating. As mentioned earlier, the streaks originating from the faces of the diamond wedge facilitate the accurate detection of the tip position during both alignment and indentation, see Fig. 13 ▸(*a*). This is a useful feature as very weak absorption of the diamond makes the tip difficult to image by, for example, intensity maps like those in Fig. 12 ▸. Furthermore, the scattered signal in the in-plane direction permits the visualization of cracks in the coating due to interface scattering from the crack faces, as shown in Fig. 13 ▸(*b*). While Meindlhumer *et al.* (2021 ▸) used the azimuthally integrated signal, we differentiate between the signal in the two orthogonal directions (in and out of the coating plane) to obtain more detailed information. The scattering from the crack is absent when the intensity in the direction parallel to the crack plane is used for mapping. No crack was observed in the unloaded condition, but the brittle nature of the coatings led to the initiation of cracks which propagated through the thickness already at the lowest load so progressive growth could be observed in the present case. Different numbers and shapes of cracks were detected for different samples and could be directly correlated to crack patterns observed by SEM after testing. Figure 13 ▸(*d*) shows a low-magnification SEM image obtained in the table-top Hitachi SU1000 SEM available at the beamline, where a single crack with morphology corresponding to Fig. 13 ▸(*b*) can be seen.

While crack formation and propagation can be readily detected also during *in situ* indentation in SEMs, the capability to visualize cracks during nanobeam stress mapping offers several exciting possibilities. Contrary to SEM, which only provides visual access to one surface of the sample, nano­diffraction probes the entire volume and allows detection even of embedded cracks invisible to the SEM. Furthermore, SEM observations do not provide simultaneous information about the stress distribution in the deformed volume, whereas the stress/strain field can be directly correlated to the initiation and propagation of crack(s) during *in situ* nanodiffraction experiments.

## 4. Summary

We have demonstrated a new nanoindenter-based sample environment for *in situ* nano- and micromechanical testing which has been commissioned at the NanoMAX beamline at MAX IV. The stability of the indenter and stage was demonstrated, which indicates that area scans of relatively large regions are feasible with sufficient scanning precision to allow step sizes of the order of the beam size. By performing *in situ* diffraction measurements with a beam size of around 60–70 nm, we show that sufficient data quality for stress mapping can be obtained even for challenging samples, such as (comparatively) large-grained, textured CVD coatings. The results obtained are very reasonable from a physical standpoint and agree well with similar studies using larger beams to map stress fields in thin coatings during indentation. Note that the analysis herein neglects several complicating effects arising from the microstructure (in particular the need for texture- and grain-morphology-dependent stress factors), but the main purpose is not accurate quantification but to demonstrate the sample environment. We also show that the method allows mapping of the plastic deformation through spatial variation in the diffraction pattern evolution and the detection of crack initiation and propagation based on the small-angle scattering intensity signal.

In summary, the state-of-the-art hard X-ray nanoprobe beamline NanoMAX at MAX IV is now equipped with an integrated sample environment allowing stress, strain and microstructure mapping capabilities where the full potential of the sub-100 nm beam can be exploited. The combination of mechanical stability and small beam size offers excellent possibilities to explore very localized strain fields/gradient or confined deformation (*e.g.* in multilayer coatings). Another benefit of the use of very small beams and fine-scanning meshes is that data can be binned post-testing to optimize the trade-off between data quality and spatial resolution. This also allows variable spatial resolution in the final maps, with finer mesh (less binning) in regions with large gradients. Although our current study exclusively demonstrates wedge indentation, it is worth noting that the indenter possesses the capability to accommodate a wide range of testing techniques, including micropillar compression, microcantilever bending and others. The modular design of the nanoindenter allows for flexible expansion, including the integration of larger load cells, dynamic testing capabilities, tensile testing, lateral force application and measurement systems, as well as the ability to conduct experiments under varied environmental conditions, such as elevated or cryogenic temperatures. Notably, this adaptable experimental setup is accessible to beamline users through the MAX IV sample environment pool.

A final point to be made regarding the use of a commercial nanoindenter intended for operation in scanning electron microscopes, or even stand-alone *ex situ*, is that this capability was maintained during the adaption to beamline use. It is therefore possible to move the sample environment into an SEM in order to perform preliminary tests to, for example, verify sample geometries or define suitable load/displacement levels and thereby optimize conditions during subsequent beam times. Furthermore, additional information can be obtained by performing identical experiments with visual access (and even digital image correlation for surface strain mapping using focused ion/electron beam deposited speckle patterns) in order to, for example, identify crack formation mechanisms, onset of slip activity or strain localization for correlation with stress/strain field evolution and/or indenter response observed during scanning nanodiffraction experiments.

## Figures and Tables

**Table 1:** Displacement drift rate, displacement noise (measured after drift correction) and force noise for the different load steps Note that the hold period duration was significantly longer for the measurement at 300 mN due to beam down-time delaying the start of the mapping.

Load	150 mN	300 mN	400 mN	500 mN
Hold period duration (min)	120	224	102	98
Average displacement drift rate (nm min^−1^)	0.34	0.02	0.09	0.24
Displacement noise (nm)	9.8	11	8.0	9.8
Force noise (µN)	7	7	7	7
