# *In situ* characterization of stresses, deformation and fracture of thin films using transmission X-ray nanodiffraction microscopy. Corrigendum

**DOI:** 10.1107/S1600577524006283

**Published:** 2024-08-06

**Authors:** Gudrun Lotze, Anand H. S. Iyer, Olof Bäcke, Sebastian Kalbfleisch, Magnus Hörnqvist Colliander

**Affiliations:** a MAX IV Laboratory, Lund, Sweden; b LINXS Institute of Advanced Neutron and X-ray Science, Lund, Sweden; c Department of Physics, Chalmers University of Technology, Gothenburg, Sweden; University of Malaga, Spain

**Keywords:** nanodiffraction, stress mapping, *in situ* deformation, nanoindentation, sample environment

## Abstract

Errors in variable subscripts, equations and Fig. 8 in Section 3.2 of the article by Lotze *et al.* [(2024). *J. Synchrotron Rad.***31**, 42–52] are corrected.

The published article by Lotze *et al.* (2024 ▸) contains a number of errors in the subscripts of variables in the text and in equations (2), (6), (7) and (8). As a consequence, some axes and labels in Fig. 8 are also incorrect. The errors originate from a change in the coordinate system used for presenting the method and results, which was made during the review stage. The error only affects the accuracy of the naming of variables, equations and labels in the article, whereas the analysis and results presented are not affected. All errors are confined to Section 3.2, and below we present a corrected version of this section, including the correct version of Fig. 8 ▸.

## 3.2. Strain and stress mapping of Ti_0.8_Al_0.2_N

A framework for analysis of the stress field during nanoindentation of thin films with the current geometry was developed by Zeilinger *et al.* (2016 ▸). Although based on several simplifying assumptions, the approach was shown to give good qualitative agreement with finite-element simulations. Here, we follow the methodology outlined by Zeilinger *et al.* (2016 ▸), briefly described below, to map the strains and stresses in the (Ti,Al)N coating in order to demonstrate the method and sample environment. We also note that for a full quantitative analysis a more complex approach, applying ψ-dependent stress factors *F*_*ij*_(ψ, *hkl*) accounting for texture, direction-dependent elastic interactions and grain morphology, is required (Welzel *et al.*, 2005 ▸). However, for the current purpose (demonstration of the sample environment capabilities) the simplified analysis is sufficient.

Figure 8(*a*) ▸ shows a typical single detector image obtained from the undeformed coating. As expected from the large grain size relative to the beam dimensions, the rings are spotty, and the intermittent azimuthal distribution shows the deposition-induced texture. To obtain the azimuthal strain distribution required for strain and stress evaluations each pattern was divided into 36 sectors (10° cakes) along the azimuthal angle (δ, see Fig. 5), which were individually reduced. For each sector the (Ti,Al)N 111 peak was fitted with a pseudo-Voigt peak shape function using the *LIPRAS* Matlab interface (version 1.466.2.0) (Esteves *et al.*, 2017 ▸), allowing calculation of the diffraction strain at each each position (*y*, *z*) from the 111 *d*-spacing, 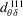, according to 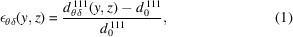
where θ is half the diffraction angle and 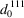 is the strain-free *d*-spacing. For an equibiaxial stress state 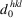 can be found when the diffraction vector satisfies 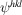 = 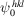, where 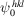 can be calculated from the plane-specific diffraction elastic constants (DECs) 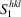 and 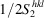. Unfortunately, DEC values are not available for the present coating, and the presence of both crystallographic texture and grain shape anisotropy significantly complicates the analysis, as mentioned above. For method demonstration, we therefore use the values obtained from the Kröner model (Kröner, 1958 ▸), as calculated by the *IsoDEC* software (Gnäupel-Herold, 2012 ▸) using single-crystal elastic constants for Ti_0.2_Al_0.8_N (Tasnádi *et al.*, 2010 ▸) (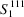 = −0.3 × 10^−3^ GPa^−1^ and 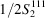 = 2.3 × 10^−3^ GPa^−1^). However, as the accuracy of the strain-free lattice parameter is critical for the strain (and hence subsequent stress) calculations, the estimation of *d*_0_ from 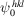 obtained under the above assumptions was not deemed suitable. Instead, 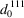 was found by an iterative procedure where the stresses found from the least-squares-fitting procedure described below yielded zero stress in the out-of-plane direction (σ_*zz*_ = 0) at the sample surface.

In the following stress analysis, we adopt the same assumptions as outlined by Zeilinger *et al.* (2016 ▸): the residual stress state in the as-deposited coating was approximated as triaxial with non-zero components σ_*ii*_ ≠ 0 and σ_*yz*_ ≠ 0 and in-plane equibiaxial stress, σ_*xx*_ = σ_*yy*_ = σ_0_. The remaining shear stress components were assumed to be negligible, σ_*xy*_ = σ_*xz*_ = 0. While it is possible to argue that other assumptions would be more suitable (*e.g.* a fully equibiaxial stress state where also σ_*zz*_ = σ_*yz*_ = 0, or full or partial relaxation of in-plane stresses due to sample preparation so that σ_*xx*_ ≠ σ_*yy*_), the main purpose of this study is to demonstrate that the data allow reliable extraction of the stresses and we therefore follow Zeilinger and coworkers in order to facilitate a direct comparison. Under the above assumptions, the relationship between the stresses and the measured strains (expressed in (ψ, ϕ) space) is given by 
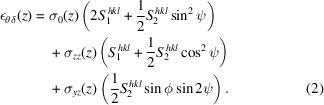
Using the following relationships, 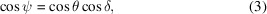

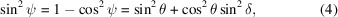

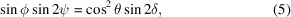
we obtain the following expression for ε_θδ_, 
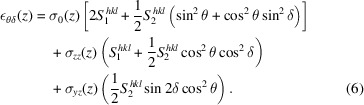
Note that equation (6) assumes no lateral variations of the stresses, and all detector images recorded at the same height (*z*) were therefore averaged before reduction and fitting to increase the statistics.

Figure 8(*b*) ▸ shows a surface plot of the azimuthally integrated intensity versus diffraction angle for a selected 2θ range as a function of position in the coating. At the bottom, the upper part of the WC–Co substrate is seen, on top of which the TiN bonding layer is visible. As seen from the normalized intensity versus *z* profiles for the (Ti,Al)N 111 and TiN 111 peaks in Fig. 8(*c*) ▸, the overlap of the signals from the two layers is of the order of 500 nm. In the following, we define *z* = 0 as the position where the TiN intensity has vanished, in order to avoid complications from the overlap region. In the case of an ideally smooth interface perfectly aligned with the beam, we would expect an overlap region corresponding to the beam size (60 nm). The interface roughness of the specific coatings investigated here was relatively small, of the order of 100 nm, which would give an overlap (contribution from both beam size and roughness) of 200 nm. Further effects arise from the interface not being completely parallel to the X-ray beam. Considering the thickness of the lamella of 40 µm, an overlap of 500 nm (accounting for the beam size contribution) corresponds to a misalignment below 1°. Further possibilities to improve the alignment of the interface with the beam through optimized sample mounting or sub-stages for isolated sample tilting should be explored in order to approach the ultimate spatial resolution defined by the beam size. In particular, this will be important for the investigation of multilayer coatings.

The iterative least-squares fitting of equation (6) to ε_θδ_(*z*) resulted in 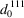 = 2.350 (7) Å [corresponding to a strain-free lattice constant *a*_0_ = 4.071 (5) Å], and the resulting residual stress profiles are plotted in Fig. 8(*d*) ▸. The in-plane stress, σ_0_, is of the order of 2.5 GPa at the (Ti,Al)N/TiN interface, and decreases to around 1.5 GPa towards the surface. This agrees well with previous reports of residual tensile stress of 1.45 GPa in as-deposited coatings (Tkadletz *et al.*, 2020 ▸). The out-of-plane stress, σ_*zz*_, is zero at the surface (as a result of the iterative fitting) while becoming tensile in the order of 1 GPa towards the (Ti,Al)N/TiN interface. This is similar to the reports by Zeilinger *et al.* (2016 ▸), who found non-zero out-of-plane stresses at the interface between the upper and lower parts of a CVD TiN coating, where the temperature had changed half-way through the deposition. They attributed this to the constraining effect of the upper part of the coating on the ability of the lower part to relax stresses during the deposition. In our case, the location of the non-zero out-of-plane component is confined to the lower half, increasing towards the (Ti,Al)N/TiN interface, and the same argument could be made. However, we note that there may be other explanations, such as possible gradients in chemistry, grain size/morphology and texture. Previous studies (Qiu *et al.*, 2020 ▸) have shown that the Ti:Al ratio is constant through the thickness, except for the innermost 200–300 nm, which is too small to explain the present gradient in σ_*zz*_. On the other hand, gradients in texture and grain morphology extend further into the coating (Qiu *et al.*, 2020 ▸), and as the (Ti,Al)N is elastically anisotropic this could potentially affect the DECs, and consequently the results of the linear regression where these values have been assumed to be constant throughout the coating. The shear stress, σ_*yz*_, is zero throughout the thickness, as expected.

Furthermore, the collection of data over the full azimuthal range allows us to compare the use of equation (6) and the standard assumption of linear *d* versus 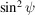 response when determining the residual stresses. Under the assumption of an equibiaxial stress state, the in-plane stress can be found from a linear fit of 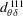 versus 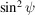 at each position (*z*),
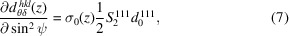
where 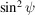 corresponding to each (θ, δ) combination is calculated using equation (4). As seen in Fig. 8(*d*) ▸, the 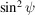 approach provides very similar values in the outer part of the coating (where the equibiaxial stress state assumption is valid) but deviates in the lower part. Again, we must remember that the here neglected complicating effects of texture, grain morphology and direction-dependent elastic interactions will also induce non-linearity in the *d* versus 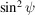 response not associated with the stress state [see *e.g.* Welzel *et al.* (2005 ▸)].

Figure 9 ▸ shows the maps in-plane (ε_*y*_) and out-of-plane (ε_*z*_) elastic strains (smoothed using a median filter with a 5 × 5 neighbourhood) during progressively increasing indentation load, as well as after complete removal of the load. Note that the top of the map does not exactly correspond to the free upper surface of the coating. The uppermost part was excluded since the surface is ‘smeared’ by surface roughness and slight misalignment, as discussed above. The strain values were obtained by averaging the strains in the ±10° sectors around the 0 and 180° and 90 and 270° positions on the detector, as shown in Fig. 8 ▸(*a*). The resulting strain fields are in good agreement with expectations. In-plane compressive strains (ε_*y*_) develop at the side of the diamond tip, whereas tensile strains develop underneath due to the cleaving effect of the sharp wedge. The out-of-plane strains (ε_*z*_) show the inverse behaviour. Small residual strains remain after unloading.

Following Zeilinger *et al.* (2016 ▸), σ_*xx*_ was assumed to be unaffected during indentation, fixed to the previously determined value of σ_0_. This assumption is necessary due to low sensitivity to strains in the *x*-direction, but also reasonable since this is the direction aligned with the diamond wedge. A benefit of the low sensitivity is that the exact value of σ_0_ is not critical, as long as it is reasonable. The stress–strain relationships are consequently given by 
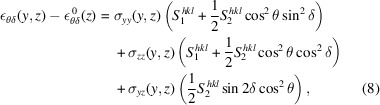
where 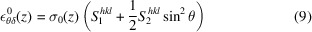
and σ_0_(*z*) is the average in-plane residual stress at *y* [as previously determined, see Fig. 8 ▸(*d*)]. From the above assumptions, the remaining non-zero stress components can then be found from weighted least-squares fitting of equation (8) at each position (*y*, *z*), where the weight corresponded to the inverse of the squared *d*-spacing error (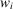 = 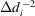). All fitting and evaluation were performed by in-house MATLAB scripts. Typical fitting results are shown in Fig. 10 ▸. Examples of the azimuthal distribution of the intensity around 2θ ≃ 19° (the 111 reflection) are shown in Figs. 10 ▸(*a*) and 10(*b*), where the stress-induced ellipticity can be seen. The corresponding 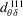-spacings for the two frames in Figs. 10 ▸(*a*) and 10 ▸(*b*) are shown in Fig. 10 ▸(*c*), together with the results from the weighted linear least-square fits of equations (6) and (8), respectively.

The evolving stress fields are shown in Fig. 11 ▸ (same regions as in Fig. 9 ▸, smoothed using a median filter with a 5 × 5 neighbourhood). Zones of very high compressive stresses in the in-plane direction (σ_*yy*_) develop at the sides of the wedge, as expected. In the out-of-plane direction a growing region with increasingly compressive stresses (σ_*zz*_) can be seen. An anti-symmetric shear stress field (σ_*yz*_) progressively develops with increasing load. As expected, most of the indentation-induced stresses are removed as the sample is unloaded, emphasizing the importance of *in situ* measurements rather than post-test stress mapping for understanding the behaviour. These results are in general agreement with previous reports of stress field development during indentation of bilayer TiN (Zeilinger *et al.*, 2016 ▸), trilayer CrN (Ecker *et al.*, 2020 ▸) and multilayer CrN–AlN (Todt *et al.*, 2020 ▸) coatings synthesized by physical vapour deposition (PVD). While these coatings had more complex multilayer structures, the small grain size and close to random crystallographic texture enable more accurate stress evaluation, consequently serving as good benchmarks. We note that the magnitude of the in-plane tensile stress decreases with increasing load, which is surprising since the cleaving effect is expected to increase. Similar trends were reported for the multilayer coating (Todt *et al.*, 2020 ▸), and attributed to the early formation of cracks which allowed relaxation of the stresses. Indeed, a crack was observed when the sample was imaged in an SEM after unloading. Additionally, the force–displacement curve [marked by the arrow in Fig. 7(*d*)] shows a small event at slightly below 100 mN, which is indicative of cracking. It is thus likely that the coating cracked before reaching a load of 150 mN. The growth of the crack explains the gradual relaxation of the in-plane stresses.

As a final note, we also investigated the effect of assuming a constant value of σ_0_ ( = 1.5 GPa, as obtained from the 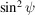 analysis) in equation (9) on the resulting stress fields during indentation, and concluded that this did not significantly affect the results.

## Figures and Tables

**Figure 8:**
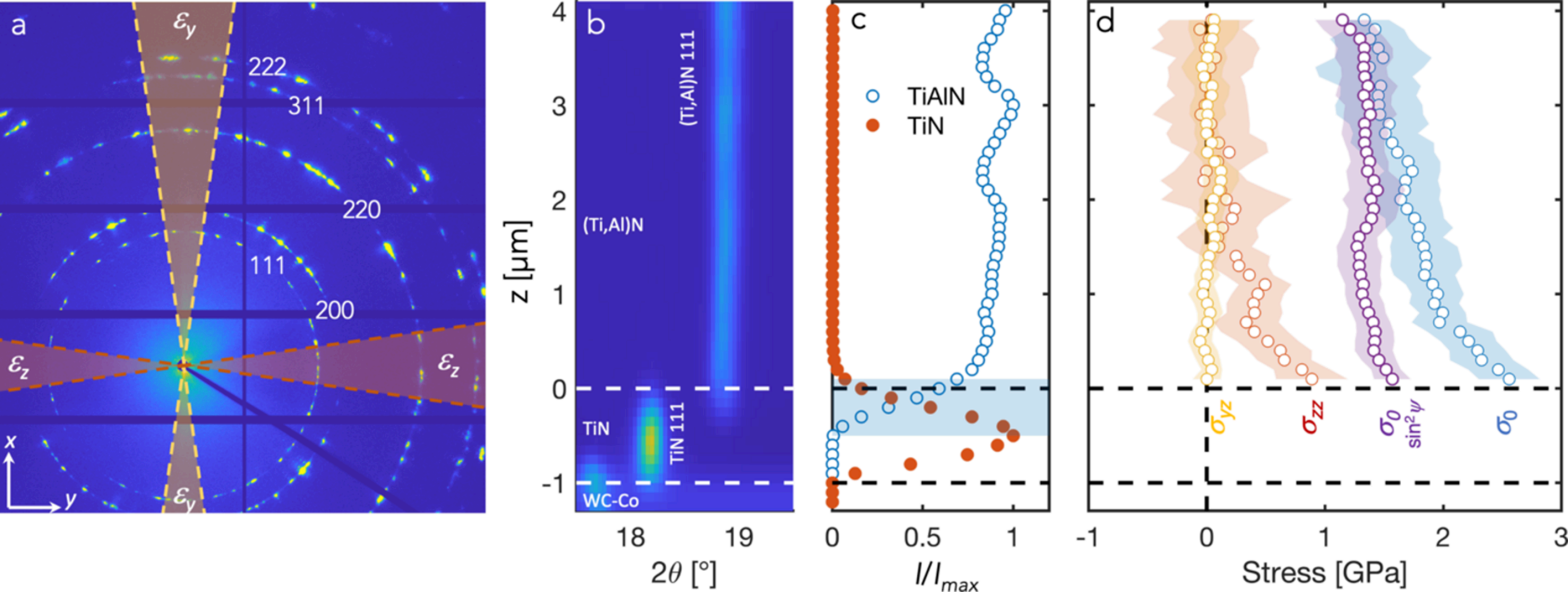
(*a*) Single detector frame from the undeformed coating. The shaded regions on the detector image correspond to the sectors used for evaluation of the in-plane (ε_*y*_) and out-of-plane (ε_*z*_) strains. (*b*) Diffractograms (intensity versus 2θ) as a function of position in the coating before application of load, showing (from bottom to top) WC–Co substrate, TiN bonding layer and (Ti,Al)N coating. (*c*) Normalized intensity distribution of the TiN and (Ti,Al)N 111 peaks with position in the coating. (*d*) Residual stresses obtained from fitting of equation (6), see text for more description. Also included is the in-plane stress calculated by the 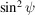 method, showing good agreement towards the top but deviations towards the (Ti,Al)N/TiN interface.

**Figure 9:**
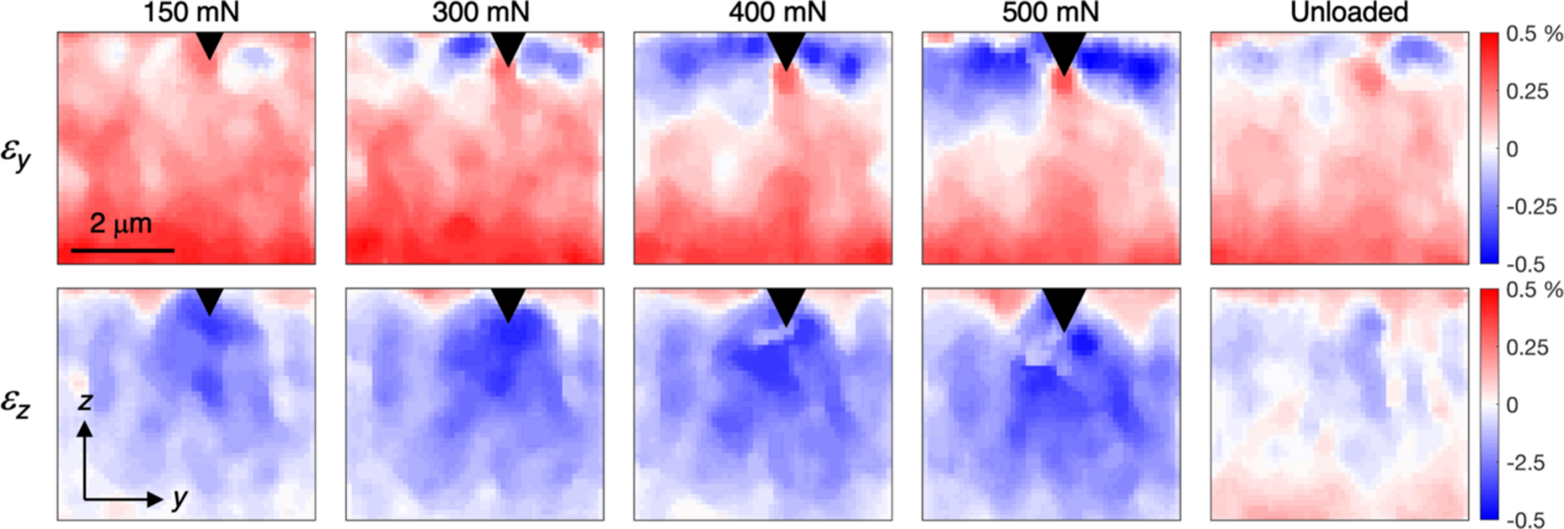
Evolution of the strain fields (ε_*y*_ and ε_*z*_) during indentation and after unloading. The black triangle indicates the approximate position of the indenter tip.

**Figure 10:**
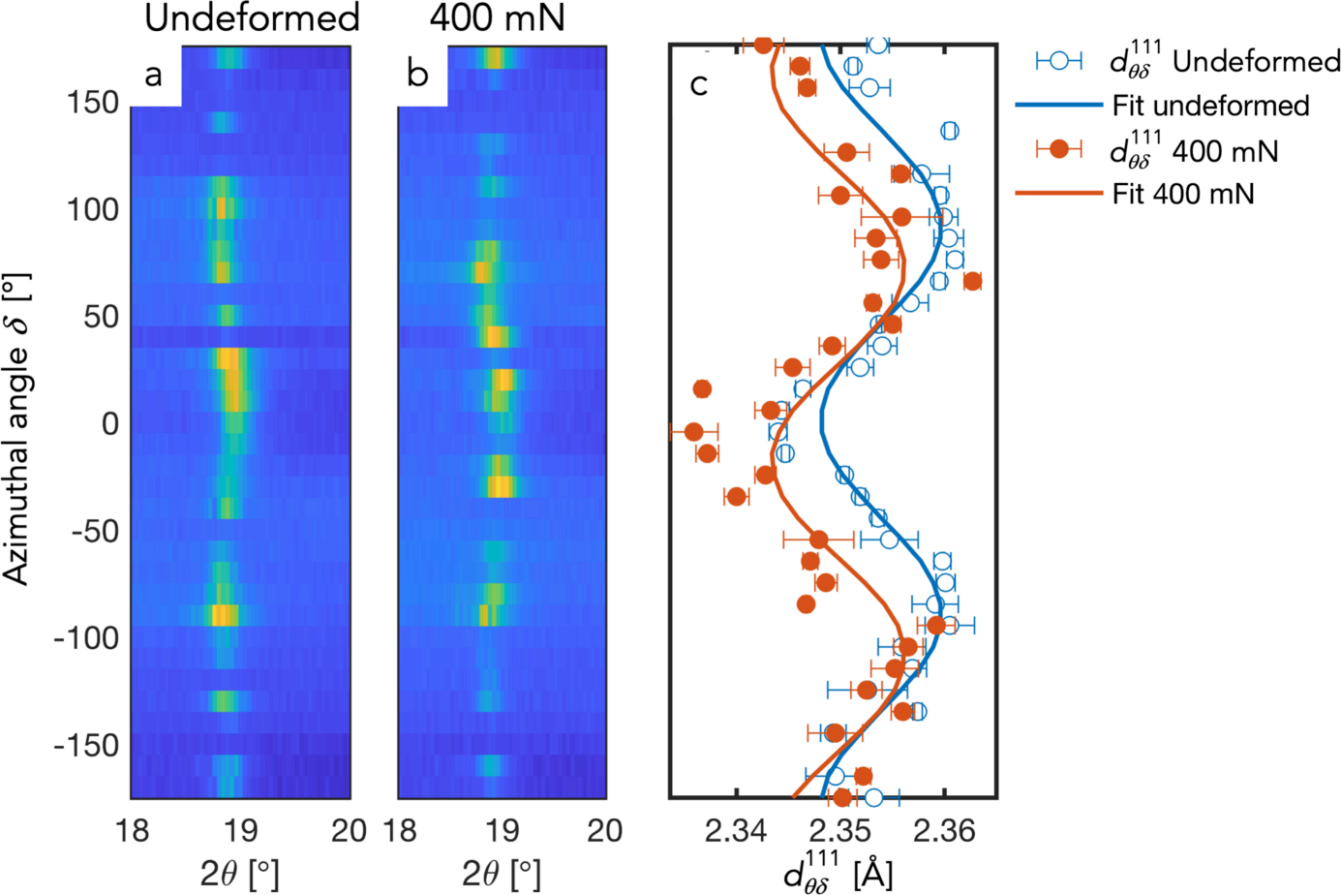
(*a* *b*) Azimuthal intensity distribution around the 111 diffraction angle from single frames taken from undeformed sample and during indentation with 400 mN load, respectively. (*c*) Fitted 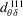-spacings and corresponding fits of equations (6) and (8) for extraction of stresses [same frames as in (*a*) and (*b*)].

**Figure 11:**
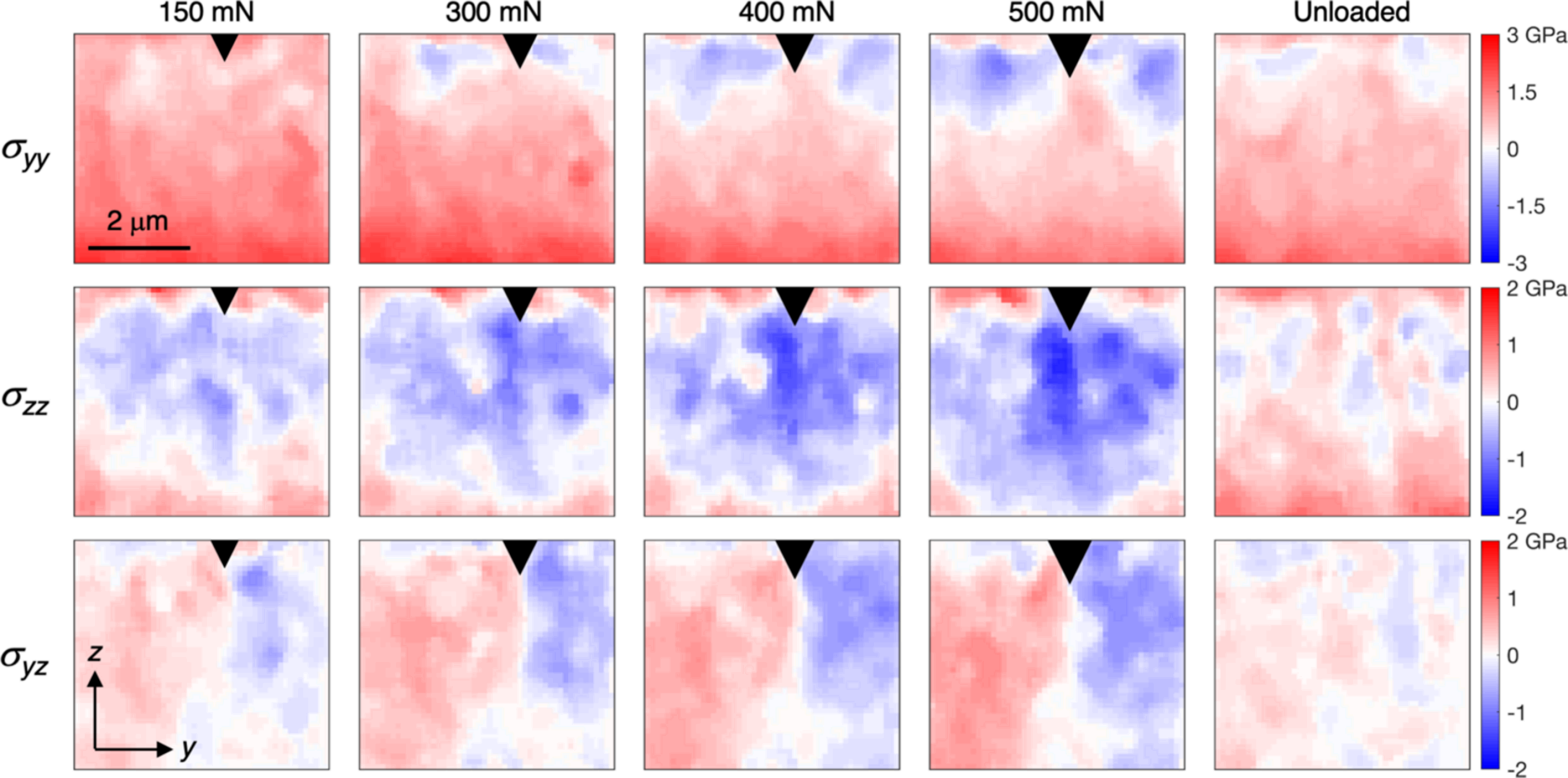
Evolution of the stress fields (σ_*yy*_, σ_*zz*_ and σ_*yz*_) in (Ti,Al)N during indentation and after unloading.
